# Reply to Dolu, K.O. Comment on “Yorulmaz et al. Enhancing the Prediction of Inborn Errors of Immunity: Integrating Jeffrey Modell Foundation Criteria with Clinical Variables Using Machine Learning. *Children* 2025, *12*, 1259”

**DOI:** 10.3390/children13040477

**Published:** 2026-03-30

**Authors:** Alaaddin Yorulmaz, Ali Şahin, Gamze Sonmez, Fadime Ceyda Eldeniz, Yahya Gül, Mehmet Ali Karaselek, Şükrü Nail Güler, Sevgi Keleş, İsmail Reisli

**Affiliations:** 1 Department of Pediatrics, Selçuk University Medical School, Konya 42250, Turkey; 2 Department of Emergency Service, Dr. Vefa Tanır Ilgın State Hospital, Konya 42600, Turkey; 3 Hacettepe University School of Medicine, Ankara 06100, Turkey; 4 Department of Pediatric Immunology and Allergy, Medicine Faculty, Necmettin Erbakan University, Konya 42140, Turkey

Response to the Letter to the Editor

We thank the author for their interest in our study and for the constructive comments regarding data consistency, feature selection, and generalizability [1,2]. We address each point below.

**Response to Comment 1: Data Consistency in Table 2**

We thank the author for drawing attention to this issue. Upon rechecking our dataset and descriptive statistics, we identified that the values “21 (7.05)” and “20 (6.71)” in the Overall column of Table 2 were inadvertently interchanged during table preparation. Correcting this typographical error by swapping these values resolves the inconsistency noted by the author. Importantly, this correction does not affect any statistical analyses, model training, or conclusions of the study.

**Response to Comment 2: Alleged Look-Ahead Bias in Feature Selection**

We appreciate the author’s comments regarding the temporal availability of certain clinical variables. However, this concern reflects a misunderstanding of the study’s intended clinical scope and methodological framework, rather than a true instance of look-ahead bias.

Our model was not designed as a point-of-first-contact screening tool, but rather as a provider-facing clinical decision support system intended to assist clinicians during the diagnostic evaluation of patients already engaged with the healthcare system. As explicitly stated in the Discussion, our predictors were not restricted to information available at the first clinical encounter, and the model was envisioned as a triage aid embedded in the electronic health record, complementing—rather than replacing—the Jeffrey Modell Foundation (JMF) warning signs and clinician judgment.

From a methodological standpoint, the inclusion of variables such as ICU admission and duration of hospitalization does not constitute look-ahead bias or temporal leakage. All predictors were defined prior to model training, and no outcome-dependent or future information was leaked across training or validation folds. The issue raised pertains to clinical timing, not statistical validity, and these concepts should not be conflated.

Clinically, the assertion that ICU admission negates the need for early warning is an oversimplification. In real-world practice, many patients with inborn errors of immunity receive a definitive diagnosis only after severe or recurrent hospitalizations, including ICU admissions. Such events are well-recognized red flags in IEI literature and were intentionally included to capture disease severity patterns that often precede or trigger diagnostic work-up.

While sensitivity analyses excluding late-stage variables may be informative for future work, their absence does not undermine the validity of the present study, nor were they within its predefined scope. Notably, limitations regarding feature availability at early encounters were transparently acknowledged in the manuscript.

**Response to Comment 3: Spectrum Bias and Generalizability**

We acknowledge the importance of spectrum bias and generalizability in predictive modeling studies; however, the interpretation presented overstates this concern and lacks appropriate clinical context.

The observed differences between the IEI and non-IEI groups—such as JMF scores and ICU admission rates—are expected and clinically appropriate given that IEI diagnoses were confirmed using European Society for Immunodeficiencies (ESID) criteria, which served as the reference standard. A cohort in which these groups substantially overlap in disease burden would raise concerns regarding case misclassification rather than improved generalizability.

Importantly, the non-IEI group did not consist of healthy controls but of symptomatic patients evaluated in a tertiary immunology clinic who failed to meet ESID diagnostic criteria. This represents a clinically relevant referral population rather than an artificially simplified control group.

Furthermore, the manuscript explicitly acknowledges the limitations of a single-center design, the absence of external validation, and potential constraints on transportability, clearly stating that the findings should be considered preliminary and that external, multicenter validation is the necessary next step. Thus, the concerns raised reiterate limitations that were already transparently disclosed rather than identify unrecognized weaknesses.

Finally, exceptionally high discrimination metrics should not automatically be interpreted as evidence of bias. In this study, performance gains were supported by rigorous nested cross-validation, repeated stratified sampling, and explainability analyses using SHAP, all of which strengthen internal validity. While external validation is essential for broader clinical adoption, its absence does not invalidate the internal findings reported.

**Concluding Statement**

In summary, while the points raised highlight important considerations for future prospective and multicenter studies, they do not undermine the methodological integrity, transparency, or clinical rationale of the present work. Rather, they align closely with the limitations and future directions already articulated in the manuscript.
